# Clinical Disease States Related to Mutations of the NOD2 Gene: A Case Report and Literature Review

**DOI:** 10.7759/cureus.38584

**Published:** 2023-05-05

**Authors:** Jasmit Walia, Rehan Mujahid

**Affiliations:** 1 Internal Medicine, St. Luke's University Hospital - Bethlehem Campus, Bethlehem, USA; 2 Allergy Immunology, St. Luke's University Hospital - Bethlehem Campus, Bethlehem, USA

**Keywords:** autoimmune like, inflammatory process, rare genetic diseases, adult-onset immunodeficiency, nod2

## Abstract

Nucleotide-binding oligomerization domain-containing protein 2 (NOD2) is a protein encoded by the *NOD2* gene and plays an important role in the immune system. NOD2 is an intracellular pattern recognition receptor (PRR) responsible for the recognition of pathogens as well as the activation of many biochemical processes within cells of the host immune system. Mutations of the *NOD2* gene can significantly impact the host's immune response against a variety of pathogens. In addition to immunodeficiency, mutations of the *NOD2* gene have also been linked with several atopic diseases and autoimmune conditions such as rheumatoid arthritis and Crohn's disease (CD). There is also a distinct set of autoinflammatory conditions that are now classified as NOD2-associated autoinflammatory diseases (NAID). We present a case of a 63-year-old female with common variable immunodeficiency, eosinophilic asthma, and rheumatoid arthritis who was found to have a *NOD2* mutation on genetic testing. As genetic testing continues to gain popularity, several disease states that were previously thought to be unrelated are now being recognized as originating from a common genetic defect.

## Introduction

Nucleotide-binding oligomerization domain-containing protein-2 (NOD2) is a protein encoded by the *NOD2* gene that plays an important role in the immune system. Mutations in the *NOD2* gene have been linked to several autoimmune and immunodeficiency diseases [1]. We present a case of a patient with a complex medical history involving multiple autoimmune, immunodeficiency, and genetic disorders who was found to have a NOD2 mutation.

## Case presentation

A 63-year-old woman was referred to our Allergy and Immunology clinic for evaluation of chronic rhinosinusitis and eosinophilic asthma. At age 13, she was diagnosed with mosaic Turner syndrome through karyotype testing, and although her adolescence and early adulthood were uneventful, she began experiencing polyarthralgia and chronic fatigue at age 40. Initially diagnosed with an undifferentiated connective tissue disorder, her condition was later re-diagnosed as seronegative rheumatoid arthritis and chronic fatigue syndrome.

During the initial evaluation, a thorough history revealed recurrent episodes of viral and bacterial infections, including Ramsay Hunt syndrome secondary to disseminated herpes simplex virus infection. Immunological workup showed suppressed levels of immunoglobulins G and A, leading to a diagnosis of common variable immunodeficiency (CVID), for which she began gamma globulin replacement therapy.

Due to multiple chronic conditions, the patient's mental and social health was severely impacted, and she had to end her nursing career prematurely. She was diagnosed with clinical depression and started on pharmacological therapy, but experienced frequent adverse reactions to multiple antidepressants due to cytochrome P450 oxidoreductase deficiency, identified through a pharmacogenetics test.

The test also revealed a heterozygous methylenetetrahydrofolate reductase (MTHFR) mutation, leading to elevated serum homocysteine levels and a diagnosis of clinical MTHFR deficiency.

Given the patient's development of multiple diseases within a short time frame, a genetic test was performed using Invitae ( San Francisco, CA, USA), which revealed a missense mutation in the *NOD2* gene, replacing an arginine with tryptophan at codon 702 of the NOD2 protein. This variant has been reported as an increased-risk allele, conferring a higher risk for autoimmune and immunodeficiency conditions and may provide a unifying diagnosis for some of her chronic conditions.

## Discussion

The *NOD2* gene, located on chromosome 16, encodes for NOD2 [1,2]. This protein is also known by other names in the literature, including caspase recruitment domain-containing protein 15 (CARD15), inflammatory bowel disease protein 1 (IBD-P1), and NOD-like receptor C2 (NLRC2).

Mutations in the *NOD2* gene have been associated with a higher risk of developing autoimmunity since its identification. It has been linked to Crohn's disease (CD), Graft versus host disease, Blau syndrome, and Yao syndrome [3]. Additionally, there have been reports of an increased risk of infections in patients with NOD2 mutations due to its effect on the host immune system [3]. The NOD2 protein actively participates in several cells of the innate and adaptive immune system [4]. Specifically, it plays an important role in the immune system's response to bacterial infections. NOD2 is a pattern recognition receptor (PRR) that recognizes bacterial components and triggers an immune response to eliminate the bacteria. It recognizes a molecule called muramyl dipeptide (MDP), which is found in the cell wall of many bacteria. When NOD2 binds to MDP, it activates a signaling pathway that leads to the production of inflammatory cytokines and antimicrobial peptides, as well as the activation of immune cells such as macrophages and dendritic cells [5].

Several types of NOD2 mutations have been identified, including missense, frameshift, and nonsense mutations. Missense mutations involve a single nucleotide change that results in a change in the amino acid sequence of the NOD2 protein. Frameshift mutations occur when an insertion or deletion of nucleotides alters the reading frame of the gene, resulting in a truncated or altered protein. Nonsense mutations involve a premature stop codon, resulting in a truncated protein that is usually nonfunctional [4].

In CD, three common NOD2 mutations have been identified: R702W, G908R, and 1007fs. These mutations are missense, missense, and frameshift mutations, respectively. These mutations are believed to impair the ability of the NOD2 protein to recognize bacterial components, leading to a dysregulated immune response and chronic inflammation [6].

Blau syndrome, another inflammatory disorder associated with NOD2 mutations, is caused by a specific missense mutation in the NOD2 gene *R334W*. This mutation alters the structure of the NOD2 protein, leading to a dysregulated immune response and chronic inflammation [7].

Other less common NOD2 mutations have been associated with various autoimmune diseases, including ulcerative colitis, psoriasis, and rheumatoid arthritis. However, the specific mechanisms by which these mutations contribute to the development of autoimmune disease are still being studied [5,6].

Overall, the types of NOD2 mutations identified vary in their impact on the protein's structure and function, and their association with specific autoimmune diseases may provide insights into the underlying mechanisms of these disorders.

Immunology

NOD2 is an intracellular PRR that recognizes MDP derived from the peptidoglycan of both gram-positive and gram-negative bacteria [5]. Pathogen-induced activation of NOD2 triggers several biochemical processes within host immune system cells. Some of the pathways affected by NOD2 include nuclear factor-κB (NF-κB) and mitogen-activated protein kinases (MAPKs). Additionally, NOD2 is involved in Caspase-1 activation, as well as the production and secretion of interleukin-1 (IL-1) and type I interferon (IFN). A malfunction in the NOD2 protein can potentially affect all these pathways, leading to clinically significant outcomes [6].

Immunosuppression

The mechanism of immunosuppression in patients with NOD2 mutations is believed to be inadequate recognition of pathogens. Peptidoglycan, the ligand for NOD2, is shared between gram-positive and gram-negative bacteria. Therefore, a mutation in this gene can interfere with the recognition of both types of bacteria, resulting in recurrent uncontrolled infections from a wide variety of microorganisms. Additionally, evidence suggests that NOD2 plays a role not only in bacterial recognition but also in viral and parasitic infections. Studies have shown that NOD2 is involved in toll-like receptor (TLR) stimulation, which is required to initiate an innate and adaptive immune response against not only bacteria but also viruses and parasites [7]. Animal models have demonstrated a decrease in NF-κB activity and a suppressed response to bacterial lipopolysaccharides, MDP, and peptidoglycan compared to the wild type (WT) [8].

Autoimmunity

Mutations in the *NOD2* gene have been found in several cases of autoimmune diseases, such as CD. NOD2 has a crucial role in the functioning of epithelial cells, and it is hypothesized that the high prevalence of CD among individuals with NOD2 mutations is due to Paneth cell malfunction. Paneth cells line the gastrointestinal mucosa and act as the first line of defense against external insults [9]. When NOD2 is changed, these cells fail to recognize bacteria effectively and permit uncontrolled growth of microbes, which can invade the intestinal lining. An abnormal immune response to these bacteria may contribute to inflammation and the gastrointestinal issues that characterize CD [9].

Additionally, NOD2 mutation is linked to a higher risk of rheumatoid arthritis. One study investigated NOD2 expression in patients with rheumatoid arthritis and found elevated NOD2 activity compared to controls. The study also detected heightened levels of proinflammatory cytokines produced by patients with NOD2 mutations [10].

Overall, the role of NOD2 in autoimmunity is complex and still being actively researched. While NOD2 is an important component of the immune system's defense against bacterial pathogens, its altered function in certain autoimmune diseases may contribute to the development of chronic inflammation and tissue damage [11]. 

Autoinflammatory Diseases

There is a distinct set of autoinflammatory conditions associated with the *NOD2* gene, which are now classified as NOD2-associated autoinflammatory diseases, also known as Yao syndrome [11]. Malfunction of NOD2 results in dysregulation of downstream signaling of the cells, contributing to aberrant inflammation and leading to the development of such autoinflammatory diseases [3].

Yao syndrome is characterized by periodic fever, dermatitis, arthritis, nonspecific gastrointestinal symptoms such as abdominal pain and diarrhea, swelling of the distal extremities, and sicca-like symptoms. The disease is recurrent and remitting, and disease flares may last a few days to weeks at a time [12].

Blau syndrome is another inflammatory disease linked to NOD2 variants, mainly presenting in the pediatric population, which can cause granulomatous dermatitis, uveitis, and inflammatory arthritis. Approximately 60% of patients with Blau syndrome develop joint deformities [12].

Atopic Diseases

Several studies have demonstrated an association between NOD2 and atopic conditions, especially asthma [13]. These studies have also found that NOD2 polymorphism is associated with elevated production of immunoglobulin E, IL-1, IL-6, and IL-8 [14,15]. The dysregulation of these proinflammatory ILs in patients with NOD2 mutations increases their susceptibility to developing atopic diseases [8].

NOD2 and Cancer

*NOD2 *is also involved in cellular homeostasis, regulating processes such as autophagy and apoptosis. Mutations in this gene can increase an individual's risk of developing cancer due to the loss of the cell's ability to recycle and regulate itself [16]. There is evidence to support a higher incidence of cancer among patients with *NOD2* mutations, including gastric, colorectal, breast, ovarian, prostate, testicular, lung, laryngeal, liver, gallbladder, biliary tract, pancreatic, small bowel, kidney, urinary bladder cancer, skin cancer, nonthyroid endocrine tumors, lymphoma, and leukemia [17].

Treatment Modalities

Although the prevalence of *NOD2* mutation-associated diseases is increasing, no clear guidelines exist for their management due to the presence of multiple different phenotypes linked to such aberrations, posing a challenge in determining the best treatment modality for each respective manifestation associated with NOD2 mutations.

In a seven-year clinical trial, 52 patients with Yao syndrome, the prototypical NOD2-associated autoinflammatory disease, were enrolled at Cleveland Clinic and systematically studied for treatment outcomes. This study demonstrated that glucocorticoids markedly decreased the disease severity and duration of flares in 19 patients (36.6%), and sulfasalazine treatment achieved a symptomatic improvement in 22 patients (42%). Additionally, three patients reported benefits with the use of canakinumab or tocilizumab, and there was no mortality during the follow-up period [18].

Furthermore, a recent study investigating the potential benefits of tumor necrosis factor (TNF) targeting therapy in Blau syndrome was conducted and published in Japan. An ex vivo study was performed using peripheral blood and induced pluripotent stem cells from 10 patients with NOD2 mutations. The study demonstrated that long-term administration of anti-TNF antibodies may correct the abnormalities that occur in the early progenitor stage by blocking the autoinflammatory loop and restoring the threshold for which IFNγ stimulation triggers an inflammatory response in macrophages [19].

Another recent study analyzed 185 patients with CD to investigate the influence of the NOD2 mutation carrier status on the response to standard medical treatments. The patients were treated with a combination of steroids (budesonide and prednisolone), immunomodulators (AZA and 6-MP), and TNFα antagonists (infliximab and adalimumab), and the response to treatment was measured by the decrease in Crohn's Disease Activity Index (CDAI) score. The results showed that patients with CD who were NOD2 carriers were less responsive to steroids but responded well to immunosuppressants. On the other hand, patients with NOD2 WT status, who were dependent on steroids, showed a significantly lower response to treatment with immunomodulators (AZA/6-MP). Furthermore, the study found that *NOD2 *variants were associated with higher levels of refractoriness to treatment with budesonide and/or prednisolone [20].

Additional studies have investigated the role of methotrexate, Janus kinase (JAK) inhibitors, anti-IL-6, and anti-IL-1 therapies in patients with NOD2 mutations, with a controversial response [18,19]. Unfortunately, further clinical trials are required to establish better treatment modalities for different disease states associated with NOD2 mutations.

## Conclusions

The *NOD2* gene plays a key role in several immune and inflammatory responses, including pathogen recognition, cellular signaling, cytokine response modulation, and apoptosis. Defects in the NOD protein can lead to various disease states, including immune deficiencies, autoimmune and autoinflammatory conditions, and atopic diseases such as asthma and cancers.

In our patient's case, the NOD2 mutation diagnosis allowed us to establish a genetic basis for several of her chronic conditions, including rheumatoid arthritis, CVID, and eosinophilic asthma. However, we could not establish a correlation between NOD2 and Turner syndrome or MTHFR deficiency.

To date, there is no known association between the *NOD2* gene and *MTHFR* gene mutations. As the use of genetic testing continues to increase, more associations between different diseases and the NOD2 gene will likely be discovered. Further research into the mechanism of *NOD2* will enable us to understand these diseases at genetic and molecular levels, potentially leading to better therapies in the future.
